# Soluble HLA measurement in saliva and cerebrospinal fluid in Caucasian patients with multiple sclerosis: a preliminary study

**DOI:** 10.1186/1742-2094-2-13

**Published:** 2005-06-02

**Authors:** Irena Adamashvili, Alireza Minagar, Eduardo Gonzalez-Toledo, Liubov Featherston, Roger E Kelley

**Affiliations:** 1 Department of Neurology, LSU Health Sciences Center, 1501 Kings Highway, Shreveport, LA 71130 USA; 2 Department of Radiology, LSU Health Sciences Center, 1501 Kings Highway, Shreveport, LA 71130 USA

## Abstract

**Background:**

Measurement of soluble HLA in body fluids has a potential role in assessing disease activity in autoimmune disorders.

**Methods:**

We applied a solid phase, enzyme-linked immunoassay to measure soluble HLA class I (sHLA-I) and class II (sHLA-II) molecules in the saliva and cerebrospinal fluid (CSF) in 13 untreated patients with relapsing-remitting form of multiple sclerosis (MS). For comparison purposes, we also studied saliva from 53 healthy subjects.

**Results:**

Saliva from normal controls had detectable sHLA-I levels in 41 of 53 individuals studied, with values ranging from 9–100 ng/ml (mean = 41 ± 2.8 ng/ml). sHLA-I was undetectable in the saliva in 11 of 13 MS patients, and in none of the CSF specimens. In contrast, mean sHLA-II concentration in the saliva of MS patients was significantly increased compared to controls (386 ± 52 unit/ml vs. 222 ± 18.4 unit/ml, t = 8.68, P < 0.005). The mean CSF sHLA-II level (369 ± 16 unit/ml) was equivalent to the mean sHLA-II concentration measured in saliva (mean = 386 ± 52 unit/ml) (P = 0.7). In patients with brain magnetic resonance imaging (MRI) enhancing lesions (n = 5), reflective of more active disease, CSF sHLA-II averaged 356 ± 26 unit/ml compared to 380 ± 51 in saliva. Similarly, in patients with non-enhancing lesions (n = 8), CSF sHLA-II averaged 377 ± 18 unit/ml compared to 390 ± 77 unit/ml in saliva. Thus, the mean sHLA-II concentration in saliva and CSF was essentially equivalent for MS patients with or without enhancing plaques.

**Conclusion:**

Our data suggest that the measurement of soluble HLA in saliva, specifically sHLA-II, correlates with the level found in the CSF. Therefore, if sHLA correlates with disease activity in MS, as has been proposed, saliva measurements provide a noninvasive correlate of CSF measurement.

## Background

The human major histocompatibility antigens, HLA, are generally cell bound, but trace amounts exist in soluble form [1-3]. These soluble HLA (sHLA) molecules may have an immunomodulatory function [4-6]. The known linkage dysequilibrium between class I and class II antigens at the cell surface may have pathophysiological significance [7]. It has been reported that the presence of soluble HLA can be explained, at least in part, by the shedding of cell bound HLA [8]. We have observed no correlation between sHLA-I and sHLA-II levels in the sera of normal individuals [9]. sHLA-I was either non-detectable, or present in very low quantities, in the urine, sweat, saliva and tears of normal individuals. sHLA-I is highly elevated in the saliva of patients with autoimmune rheumatic diseases [2,10]. sHLA-II is routinely detectable in the urine, tears, sweat and saliva of normal individuals, but concentrations of sHLA-II are not observed to be elevated in rheumatological diseases [10,11].

In the neurological realm, there is a possible alteration of sHLA-I and/or sHLA-II levels as a reflection of disease activity in multiple sclerosis (MS). Clinical and brain magnetic resonance imaging (MRI) disease activity in MS is associated with fluctuations in sHLA-I and sHLA-II levels in the serum and cerebrospinal fluid (CSF) of patients with MS [12-14]. However, the published reports are somewhat in conflict. There has been reported elevation of serum sHLA-II, but not of serum sHLA-I, and an increase in CSF sHLA-I, but not CSF sHLA-II concentrations, in patients with MS [12,13]. However, an elevation of CSF sHLA II and I as well as an increase in serum sHLA-I, but not in serum HLA-II levels, in MS has been reported [14]. Fainardi et al [15] reported a decrease in sHLA-I concentrations during exacerbations in MS, but an increase in CSF sHLA-I was observed in patients with lesional activity by MRI brain scan. The variability in the studies, to date, could possibly be explained by variability in phenotypic expression in genetically susceptible individuals as well as in assay methodology. Recent studies have demonstrated that variations in sHLA concentrations are due, at least in part, to the HLA allospecificities [16-18]. Racial-ethnic factors may also have an influence on sHLA levels [18,19]. Thus, it appears advantageous to assess sHLA measurements in subjects with a similar racial-ethnic background.

Theoretically, we would expect that measurement of sHLA in CSF would be most likely to reflect central nervous system (CNS) disease activity if indeed such measurement could serve as a monitor of a disorder such as MS. However, CSF exams are invasive and not without potential complications. Therefore, we sought to determine whether more readily accessible body fluid, specifically saliva, might provide correlative sHLA measurements in an autoimmune-mediated CNS disease such as MS.

## Methods

We analyzed CSF and saliva from thirteen consecutive Caucasian patients with relapsing-remitting form of MS (RRMS) defined by the McDonald criteria [20]. None of these patients was on immunomodulating therapy for at least six months prior to entrance into the study. We also studied saliva from fifty-three healthy subjects with no history of autoimmune disease for the purpose of comparison. Because there is a high degree of racial variation in the gene frequencies of HLA [7], we limited study participation to Caucasians born in the United States and residing in Louisiana.

Saliva samples were collected through expectoration preceded by rinsing of the mouth with sterile water. The resultant salivary samples were collected into test tubes and stored at -20°C until subsequent assay. CSF was collected by standard sterile lumbar puncture technique after the informed consent was reviewed with the patient and signed.

Brain MRI was performed using a 1.5 T machine with a standard quadrature head coil. The imaging protocol included sagittal T1-, axial T1-, T2-weighted, and fluid attenuated inversion recovery (FLAIR) images. All MRI scans were performed before and after (Gd-DTPA) infusion. Axial T2-weighted and pre- and post-contrast T1-weighted images were used for assessment of MS plaques. The images were independently interpreted using inspection and computer-assisted techniques by a neuroradiologist. Detection of lesions, compatible with MS, was made by visual inspection as was determination of the absence or the presence of contrast enhancing lesion. Computer based software allowed comparison of the lesions among different groups. Comparisons were made between all 13 MS patients who were subgrouped into either those with and those without enhancing plaques in their brain MRI scans.

Hybridoma cell lines W6/32 (anti-HLA-A, B, C), L368 (anti-human B_2_-microglobulin), Ab2.06, L203 and IVA-12 (anti-HLA-DR) were obtained from the American Type Culture^® ^collection (Rockville, MD). These lines were expanded and the mAbs were produced in BALB/c mice as described previously [21]. Anti-class I HLA-monoclonal antibody W6/32 detects a common determinate on the a-chain of all HLA class I molecules. Monoclonal antibody L368 detects B_2_-microglobulin, which is a constituent of all HLA class I molecules [22,23]. Anti-class II HLA-monoclonal antibodies Ab2.06, L203 and IVA-12 react with non-competing epitopes in the constant domain of HLA-DR molecules [24-26].

The solid-phase ELISA for sHLA-I has been previously described [3,10,16,21]. The levels of sHLA-II were determined using a previously described assay [9,11] with minor modifications. Briefly, test samples were added to appropriate wells containing an anti-Class I (W6/32) or anti-Class II (Ab2.06) monoclonal antibody (Mab) coated beads. The reaction proceeded for 30 minutes for sHLA-I and for two hours for sHLA-II at 45°C. The beads were then washed × 3 with distilled water and 200 νl of peroxidase-labeled anti-B_2_M monoclonal antibody (L368) for sHLA-I or L 2.03 Mab for sHLA-II were added to each bead and incubated for an additional hour at 45°C. After additional washes, the color reaction was started by adding the appropriate substrate. Absorbance was measured at 492 nm.

Each assay included a standard curve derived from positive and negative controls. Negative controls consisted of 2% BSA and human serum, free of sHLA-I and sHLA-II. Positive control standards were prepared by chromatography of pooled serum over a CL-6B Sepharose Mab W6/32 gel column. The sHLA-I captured by the Mab column was eluted with glycine HCL buffer (0.1 M glycine, pH 2.5). Fractions rich in sHLA-I were neutralized immediately with dibasic sodium phosphate pooled and dialyzed against saline. Total protein was quantitated with the Pierce BCA kit^® ^(Rockford, IL), which was assumed to be pure sHLA-I. With each assay, a standard curve was established by including, in duplicate, seven sHLA-I standards (100, 50, 25, 12.5, 6.25, 3.1 and 0 ng/ml) of pure sHLA-I protein. The test values were calculated from the curve described by these standards. All sHLA-I assays were standardized with dilutions of banked standard serum. Measurements were reproducible.

For sHLA-II values, the wide range of sHLA-II concentrations reported from studies of serum of normal individuals [3,9,17,26-30] seem likely to reflect the use of various standards or to the different characteristics of the monoclonal antibodies used in described techniques. Although initial standardization of the sHLA-II assay has been made previously and reported by us [9], in this study, for greater precision of analysis, the amount of sHLA-II was inferred directly from the ELISA absorbance value (OD) within each sample of body fluid tested. The OD of studied samples corresponding to sHLA-II values were compared with the OD values of 5% BSA that had been utilized as dilution factor and negative control within each procedure.

Comparisons of mean values for sHLA in study subjects and controls were made with the two-tailed t-test for the means of independent samples. However, this only applied for sHLA-II measurements in our study. P values < 0.05 were considered significant.

## Results

All normal individuals tested had measurable amounts of sHLA-II in the saliva with a range of 186–362 unit/ml and a mean of 222 ± 18 unit/ml (Table-1). In saliva, sHLA-I levels ranged from 0.86 to 100 ng/ml. In five subjects, measurements were below the sensitivity of the assay and thus were non-detectable. These results are in agreement with our previous measurements of sHLA in normal saliva where we found that seven of thirty-seven subjects did not have detectable levels of sHLA-I in this body fluid [11]. However, in this study we raised the question as to whether these individuals represent a population with no sHLA-I. This is apparently not the case, as all saliva samples (n = 13) that were passed over a monoclonal antibody w6/32 column yielded the presence sHLA-I, regardless of detectability by assay. Thus, sHLA-I is present in the saliva in some quantities, however these values are too low to be distinguished from zero in the test system.

**Table 1:** Concentrations of sHLA-II in cerebrospinal fluid (CSF) and saliva in multiple sclerosis patient subgroups and controls (unit/ml)

		CSF	Saliva		
Study group	Number	Mean ± std dev	Mean ± std dev	t-value	p-value

RRMS-total	13	369 ± 16	386 ± 52	.70	.70
RRMS-C(+)	5	356 ± 26	380 ± 51	.44	.67
RRMS-C(-)	8	377 ± 18	390 ± 77	.56	.59
controls	52	----	222 ± 18	8.68	<.0005*

RRMS = relapsing remitting multiple sclerosis

(+) = contrast enhancement by MRI of brain (active plaque formation)

(-) = without contrast enhancing plaques by MRI (inactive)

*Comparison of saliva sHLA-II in patients (386 ± 52) vs. controls (222 ± 18)

**Figure 1:**
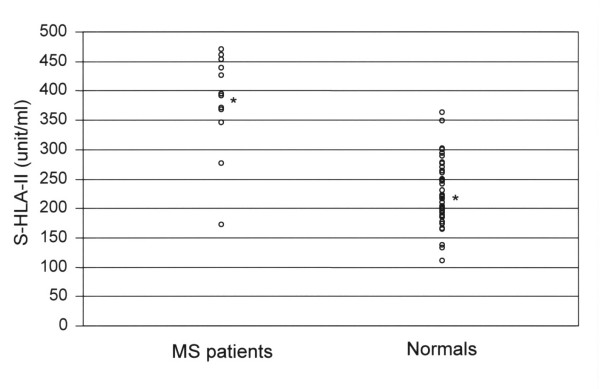
Demonstration of soluble HLA class II levels in the saliva of multiple sclerosis patients versus controls. The mean ( ± S.D.) values, denoted by *, are 386 ± 52 unit/ml for patients and 222 ± 18 unit/ml for controls (P < .0005).

**Figure 2:**
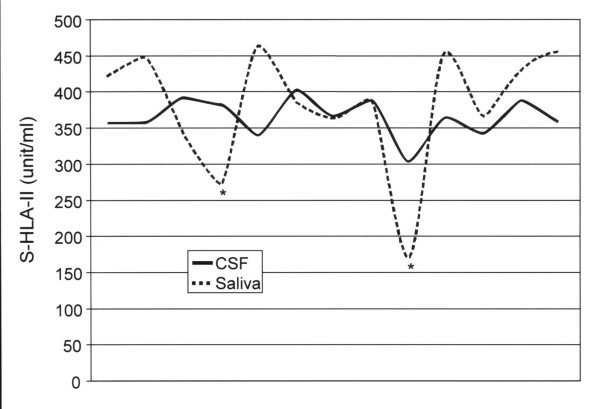
Demonstration of the distribution curves for soluble HLA-II levels in the cerebrospinal fluid (unbroken line) and saliva (dotted line) in multiple sclerosis patients. Despite two outlying values, denoted by *, there is a fairly equivalent distribution with a mean sHLA-II concentration of 369 unit/ml for cerebrospinal fluid and 386 unit/ml for saliva (t = -.70, P = 0.7)

For the 13 patients with RRMS, two had a relatively low concentration of sHLA-II (172 unit/ml and 276 unit/ml, respectively) in saliva, while the remaining eleven had relatively high amounts of sHLA-II, ranging from 329 unit/ml to 470 unit/ml with a mean value of 386 ± 52 unit/mL (Table-1). This value was highly significant when compared to those of normals (t = 8.68, P < .0005) (Figure 1). Of interest, each patient with RRMS had elevated levels of sHLA-II in the CSF, with a mean of 369 unit/ml, and this was essentially equivalent to the mean sHLA-II concentration in saliva (mean = 386 unit/ml, t = -.70, P = 0.5). In addition, it was noted that CSF and saliva sHLA-II distribution curves were fairly equivalent, except for two outliers (Figure 2). sHLA-II concentrations in the CSF and saliva of MS patients were further analyzed by subgrouping them into those with enhancing lesions vs. those without enhancing lesions on brain MRI, with the understanding that contrast enhancement tends to reflect disease activity. Comparison of sHLA-II concentrations in the patients with enhancing lesions (N = 5) to the patients with non-enhancing lesions (N = 8), revealed no significant CSF (356 vs. 377 unit/ml, t = 1.49, P = 0.16) or saliva (380 vs. 390 unit/ml, t = 0.2, P = 0.84) differences.

The measurements of the saliva and the CSF HLA-I demonstrated the following: sHLA I was highly elevated in the CSF and saliva of only two patients with RRMS, during an exacerbation (mean = 854 ng/ml), while the remaining eleven patients had no detectable sHLA-I in CSF or saliva. This was also true for those patients with or without contrast enhancement by MRI brain scan.

## Discussion

There is considerable interest in the apparent ability of HLA complex to release molecules, identified as sHLA proteins, into the surrounding fluids as this may translate into a biological monitor of autoimmune disease activity. However, the pathways responsible for, and the potential pathophysiological significance, of sHLA material in different body fluids have not been determined. Active secretion of sHLA-I by liver cells and activated immunocompetent cells [31,32] are suggested sources for its production in serum. A small number of studies have shown that sHLA-I molecules appearing in serum are heterogeneous in molecular mass and multiple molecular forms of sHLA may have different physiological roles [33-35]. It has been proposed that sHLA-I can appear in the serum as a result of shedding from the cell membranes, can be a product of proteolysis, or can be secreted by an alternative splicing pathway [8,33,36]. It is possible that serum HLA-II may be derived from similar processes. However, there is no supportive data for this assumption.

Biochemical studies of sHLA-II in the synovial fluid of patients with rheumatoid arthritis revealed a preferential release of high-molecular-weight (1000 kDa) sHLA-II in the inflamed synovium, but not in the serum [27]. In addition, attempts to induce production of similar material from a cell line expressing HLA-II on a cell surface have failed, indicating that release of sHLA-II is an active process. Of interest, sweat has been shown to possess polymorphic structures identical to those of serum HLA-I. However, excretion of sHLA-I in sweat has been found to be in markedly lower quantities than in serum [11,38]. We reported the occurrence of 39 kDa sHLA-I in saliva as well as in serum during active Sjögren's disease and systemic lupus erythematosus, and the presence of 35–37 kDa HLA-I in both body fluids when the disease was relatively inactive [10,35]. Taken together, it appears that the presence of sHLA in different body fluids has physiological relevance. However, it remains to be determined in which body fluids sHLA production reflects immunoreactivity, if indeed this is the case.

We reported a substantial elevation of saliva sHLA-I in patients with autoimmune rheumatic diseases, when the saliva sHLA-II concentrations were in normal range [10]. sHLA-I concentrations in saliva were observed to be related to the activity or clinical course of rheumatological diseases. The present study indicates correlative elevation of sHLA-II in the saliva and CSF of patients with RRMS. Of particular interest, the great majority of sHLA-II measurements were equivalently distributed in both body fluids. In contrast, sHLA-I was undetectable in most specimens, with only occasional elevation, possibly associated with some sub-clinical episodes of the disease. It is possible that sHLA-I and sHLA-II are selectively altered by the immunological process and are preferentially impacted by different pathological mechanisms.

The differential expression of sHLA concentrations observed in this study requires further investigation to determine if this is directly related to immune responsiveness or is an epiphenomenon of the pathogenetic process. In a recent study, an increase in serum sHLA-I in MS patients treated with interferon beta 1b was reported, and the elevation correlated with response to therapy [39]. However, whether sHLA from other body fluids follows a similar pattern remains to be determined as evidenced by the reciprocal relationship between CSF and serum sHLA-I levels in MS reported by these same investigators [15].

It appears from this preliminary study that sHLA-II is the predominant class of sHLA molecules present in the CSF and saliva of MS patients. This is in contrast to CSF and saliva sHLA-I, which we have found to be in non-detectable quantities. The reduced sHLA-I and augmented sHLA-II observed in these body fluids may reflect the active stage of RRMS, triggered by the stimulation of immune system in the absence of immunosuppressive therapy. Our results indicate that measurement of saliva sHLA-II may be a potential noninvasive biological marker of disease activity in a primary CNS disease such as MS.

## Competing interests

The author(s) declare that they have no competing interests.

## Authors' contributions

Dr. Adamashvili has contributed to this manuscript by providing expertise for laboratory measurement of soluble HLA-I and soluble HAL-II.

Drs. Minagar and Kelley have contributed to this manuscript by recruiting and examining multiple sclerosis patients, interpretation of data and preparing the manuscript.

Dr. Gonzalez-Toledo has contributed to this manuscript by interpreting the neuro-radiology studies and generating neuro-radiology data.

Dr. Featherston has contributed to this manuscript by doing statistical analysis and generating the figures.
